# Anti-Inflammatory and Protective Effects of *Juncus effusus* L. Water Extract on Oral Keratinocytes

**DOI:** 10.1155/2022/9770899

**Published:** 2022-01-04

**Authors:** Akihiro Wada, Keiji Murakami, Yumi Ishikawa, Takashi Amoh, Kouji Hirao, Yuki Hosokawa, Daisuke Hinode, Yoichiro Miyake, Hiromichi Yumoto

**Affiliations:** ^1^Department of Periodontology and Endodontology, Institute of Biomedical Sciences, Tokushima University Graduate School, Tokushima, Tokushima, Japan; ^2^Department of Clinical Nutrition, Faculty of Health Science and Technology, Kawasaki University of Medical Welfare, Kurashiki, Okayama, Japan; ^3^Department of Oral Health Sciences, Faculty of Health Sciences, Osaka Dental University, Osaka, Japan; ^4^Department of Dental Hygiene, Mejiro University College, Tokyo, Japan; ^5^Department of Regenerative Dental Medicine, Institute of Biomedical Sciences, Tokushima University Graduate School, Tokushima, Tokushima, Japan; ^6^Department of Hygiene and Oral Health Science, Institute of Biomedical Sciences, Tokushima University Graduate School, Tokushima, Tokushima, Japan; ^7^Department of Oral Health Sciences, Faculty of Health and Welfare, Tokushima Bunri University, Tokushima, Tokushima, Japan

## Abstract

Periodontitis is a chronic inflammatory disease caused by periodontopathogenic bacteria that form biofilms in periodontal pockets. The gingival epithelium acts as the first physical barrier in fighting attacks by periodontopathogenic pathogens, such as the primary etiological agent *Porphyromonas gingivalis*, and various exogenous chemicals, as well as regulates the local innate immune responses. Therefore, the development of novel oral care products to inhibit inflammatory reactions caused by bacterial infection and protect the gingival epithelium is necessary. *Juncus effusus* L. has generally been used as an indigenous medicine, such as a diuretic, an antipyretic, and an analgesic, in ancient practice. In this study, we examined the effects of a water extract from *J. effusus* L. on the inhibition of the inflammatory reaction elicited by bacterial infection and protection of the oral epithelium by chemical irritation. Pretreatment of oral epithelial cells with the water extract from *J. effusus* L. significantly reduced *P. gingivalis* or its lipopolysaccharide- (LPS-) mediated production of chemokines (interleukin-8 and C-C-chemokine ligand20) in a concentration-dependent manner with comparable to or greater effects than epigallocatechin gallate and protected oral epithelial cells from injury by chemical irritants, cetylpyridinium chloride, and benzethonium chloride. Moreover, the water extract from *J*. *effusus* L. in the presence of antimicrobial agents or antifibrinolytics already used as ingredients in mouthwash could significantly reduce the production of chemokines from *P. gingivalis* LPS-stimulated oral epithelial cells in a concentration-dependent manner. These findings suggest that the water extract from *J*. *effusus* L. is potentially useful for oral care to prevent oral infections, such as periodontal infections, and maintain oral epithelial function.

## 1. Introduction

Periodontitis is a chronic inflammatory disease caused by periodontopathogenic bacteria that form biofilms in the periodontal pocket, leading to the destruction of the supporting tissues of the teeth and finally tooth loss [1]. Gingival epithelial cells play an important role as the first physical defense against bacterial invasion and orchestrate the local innate immune response against colonizing pathogens, such as *Porphyromonas gingivalis*, a primary etiological agent [2]. The gingival epithelium and epithelial cells differentially express Toll-like receptors (TLRs; TLR1 to TLR10), which are pattern recognition receptors (PRRs) involved in sensing bacteria and various products called pathogen-associated molecular patterns (PAMPs) [3]. Among these PRRs, TLR2 is prominently expressed in the epithelium of periodontal-diseased pockets and activates the downstream signaling pathways involved in the production of chemokines, such as interleukin- (IL-) 8, by its specific ligands, including the *P. gingivalis* lipopolysaccharide (LPS) [4, 5]. Moreover, a previous report demonstrated that TLR4 immunoreactivity was found in healthy gingival epithelium in vivo, and TLR4 expression was also detected in keratinocytes in vitro [6]. Regarding the triggering and enhancement of acute inflammation, IL-8, produced early in the inflammatory response, has a pivotal role in the recruitment and activation of neutrophils, which are important effector cells involved in innate and acquired immunity during inflammatory diseases [5, 7–9]. The C-C-chemokine ligand (CCL)20, which exhibits antimicrobial activity against Gram-negative bacteria [10, 11], is also a chemokine that links innate and adaptive immune responses by chemoattracting and activating immature dendritic cells [12]. Our previous study also demonstrated that inflamed gingival epithelial cells in tissues with periodontal disease were positive for CCL20 [13]. Therefore, the development of a therapy to suppress the overproduction of proinflammatory mediators, including IL-8 and CCL20, would be advantageous.

*Juncus effusus* growing in marshes in Japan, China, Taiwan, and North America has been commonly used for over 1000 years as a raw material for making tatami mats and is also used as a food additive because its powder has been confirmed to be highly safe. Moreover, it has been reported that *Juncus effusus* L. has generally been used as an indigenous medicine, such as a diuretic, an antipyretic, and an analgesic, in ancient practice for a long time. However, the detailed mechanisms underlying these effects have not yet been clarified. Recent in vitro and in vivo studies have reported the anti-inflammatory effects of ethanol extract from the pith of *J*. *effusus* in LPS-stimulated murine macrophages and in a mouse edema model [14]. Another study also demonstrated that the compounds isolated from the ethanolic extract of the medullae of *J*. *effusus* exhibited moderate anti-inflammatory activity in LPS-stimulated murine macrophages [15]. Although ethanol is still a component of a considerable number of oral care products, such as mouthwashes, a review article concluded that consumption of ethanol and its metabolite, acetaldehyde, increases the risk of oral cancer, and evidence supporting the carcinogenicity of ethanol-containing mouthwashes is limited, suggesting that other compounds can easily be substituted for ethanol in mouthwashes [16]. In this regard, reports have shown that alcohol-free mouthwashes are as effective as their alcohol-containing counterparts [17, 18], and being alcohol-free makes them nonirritating and gentle to use in persons with sensitive or inflamed mucosa, such as immunocompromised and cancer patients, significantly decreasing the incidence of other adverse events [19, 20].

Moreover, oral care products are being developed for new or multiple benefits of oral health and have become complex due to the presence of active ingredients and excipients. Cetylpyridinium chloride (CPC) and benzethonium chloride (BTC) are synthetic bactericidal cationic quaternary ammonium surfactants that are widely used as topical anti-infective agents and disinfectants in mouthwashes and toothpastes because of their effectiveness in preventing dental plaque accumulation and reducing gingival inflammation [21, 22]. However, CPC has been well characterized as a typical skin and corneal epithelial irritant, whereas BTC is cytotoxic to normal human oral keratinocytes [23–25]. Concentrated detergents, such as CPC and BTC, are generally considered destructive to mucous membranes and cause a burning sensation with irritation in the mouth.

Newly developed oral care products that are ethanol-free and can inhibit the subsequent innate immune reaction and protect against oral epithelial irritation are therefore required. This study investigated the potential of a water extract from *J. effusus* L. in preventing periodontal inflammation and protecting the oral epithelium. We first investigated the effects of the water extract from *J*. *effusus* L. on the production of IL-8 and CCL20 induced by LPS from *P. gingivalis* and *Escherichia coli* which are TLR2 and TLR4, respectively. The protective effect of the water extract from *J*. *effusus* L. against chemical irritation to oral epithelial cells and its anti-inflammatory effects in the presence of antimicrobial agents or antifibrinolytics as ingredients in mouthwash were further examined in this study.

## 2. Materials and Methods

### 2.1. Reagents

*P*. *gingivalis* LPS and Pam3CSK4 as TLR2 ligand and ultrapure *E*. *coli* LPS as TLR4 ligand were purchased from InvivoGen (San Diego, CA, USA). Epigallocatechin gallate (EGCG), CPC, BTC, and tranexamic acid (TA; C_8_H_15_NO_2_) were purchased from Sigma-Aldrich (St. Louis, MO, USA). Fluorescence-labeled Pam3CSK4 was obtained from GMC Microcollection (Tübingen, Germany).

### 2.2. *J. effusus* L. Water Extract

*J. effusus* L. powder, named “igusa vegetable powder,” was kindly provided by Inada Limited Company (Yatsushiro, Kumamoto, Japan). *J. effusus* L. powder (10 g) was added to 100 mL of ultrapure water and extracted at 60°C for 5 h. After centrifugation at 1200 × *g* for 15 min, the supernatant was filtrated through a 0.45 *μ*m filter. The filtrate, condensed to 20 times the concentration rate under diminished pressure, was finally used as the undiluted *J. effusus* L. water extract.

### 2.3. Cell Culture

RT-7 cells, an immortalized human keratinocyte cell line, kindly provided by Dr. N. Kamata (Hiroshima University, Hiroshima, Japan) as described previously [26] were cultured in Keratinocyte SFM (Gibco BRL, Gaithersburg, MD, USA) supplemented with 100 U/mL penicillin and 100 *μ*g/mL streptomycin (Gibco BRL) at 37°C in a water-saturated atmosphere of 95% air and 5% CO_2_. Confluent monolayers were cultured with *P. gingivalis* LPS, Pam3CSK4, or ultrapure *E. coli* LPS and/or diluted *J. effusus* L. water extract or EGCG for the indicated period in each experiment.

### 2.4. Enzyme-Linked Immunosorbent Assay (ELISA)

The concentrations of IL-8 and CCL20 in cell culture supernatants were determined using an ELISA kit (R&D Systems, Minneapolis, MN, USA) according to the manufacturer's instructions.

### 2.5. Chemokine Antibody Array

Chemokine detection in cell culture supernatants obtained from RT-7 cells treated with *P. gingivalis* LPS and/or the *J. effusus* L. water extract was performed using the Human Chemokine Antibody Array 1 (Ray Biotech, Inc., Norcross, GA, USA), according to the manufacturer's instructions.

### 2.6. Binding of the Fluorescence-Labeled TLR2 Ligand to Oral Keratinocytes

The RT-7 monolayer pretreated with the *J. effusus* L. water extract for 3 min was reacted with fluorescence-labeled Pam3CSK4 (1 *μ*g/mL) for 2 h at 37°C. After washing with phosphate-buffered saline (PBS), the fluorescence level of Pam3CSK4 bound to RT-7 cells was measured with excitation at 485 nm and emission at 535 nm using a fluorescence microplate reader (Twinkle LB 970; Berthold Technologies, Bad Wildbad, Germany) [27].

### 2.7. *J. effusus* L. Water Extract Protection Assay

RT-7 monolayers cultured in a 24-well plate were treated with diluted *J. effusus* L. water extract and 0.005% CPC or BTC for 3 min at 37°C, and then, the culture medium was removed. Fresh medium was added, and RT-7 monolayers were cultured at 37°C for 24 h. For the cytotoxicity assay, the levels of lactate dehydrogenase (LDH) in recovered cell culture supernatants were determined using an LDH cytotoxicity assay kit (Cayman Chemical Co., Ann Arbor, MI, USA) [27].

### 2.8. Statistical Analysis

All statistical analyses were performed using unpaired Student's *t*-test. Differences were considered significant when the probability value was less than 5% (*P* < 0.05).

## 3. Results

### 3.1. Effects of *J. effusus* L. Water Extract on IL-8 Production in *P. gingivalis* LPS-Stimulated Oral Keratinocytes

**Figure 1:**
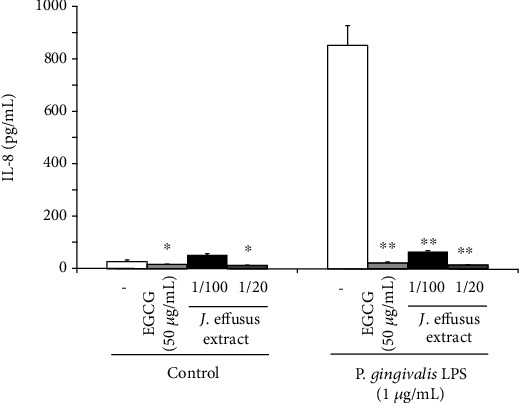
Effects of the *Juncus effusus* water extract on interleukin-8 (IL-8) production in *Porphyromonas gingivali*s lipopolysaccharide- (LPS-) stimulated oral keratinocytes. RT-7 cells were cultured with the *J. effusus* water extract (20- or 100-fold dilution) or epigallocatechin gallate (EGCG) (50 *μ*g/mL) and *P. gingivalis* LPS (1 *μ*g/mL) for 24 h. The IL-8 level in culture supernatants was determined by enzyme-linked immunosorbent assay and expressed as the mean ± standard deviations of three independent experiments. ^∗^*P* < 0.05 and ^∗∗^*P* < 0.01 compared to that in the control (no treatment with the *J. effusus* water extract or EGCG).

*P. gingivalis* LPS increases the production of proinflammatory cytokines, including IL-8, through the TLR2/TLR1-dependnent pathway in oral epithelial cells [28–30]. It has been reported that EGCG, the most predominant polyphenol in green tea, possesses various pharmacological activities [31]. With respect to its anti-inflammatory properties, EGCG can downregulate the expression of various inflammatory mediators in *P. gingivalis* LPS-stimulated human periodontal ligament stem cells [32] and significantly reduce the secretion of various inflammatory mediators, such as IL-8, in a three-dimensional coculture model of gingival epithelial cells and fibroblasts stimulated with LPS from *Aggregatibacter actinomycetemcomitans*, which is strongly associated with aggressive periodontitis [33]. We initially examined whether the *J. effusus* L. water extract could inhibit *P. gingivalis* LPS-induced IL-8 production in RT-7 cells and compared its efficacy with that of EGCG. Coincubation with the 100-fold diluted *J. effusus* L. water extract effectively reduced the production of IL-8 in *P. gingivalis* LPS-stimulated RT-7 cells, and the efficacy of its 20-fold diluted extract was comparable to that of EGCG at 50 *μ*g/mL (Figure 1).

### 3.2. Effect of *J. effusus* L. Water Extract Pretreatment on IL-8 Production in *P. gingivalis* LPS-Stimulated Oral Keratinocytes

**Figure 2:**
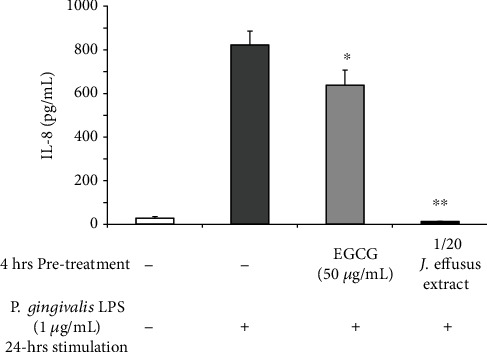
Effects of *Juncus effusus* water extract pretreatment on interleukin-8 (IL-8) production in *Porphyromonas gingivalis* lipopolysaccharide- (LPS-) stimulated oral keratinocytes. RT-7 cells were pretreated with the *J. effusus* water extract (20-fold dilution) or epigallocatechin gallate (EGCG) (50 *μ*g/mL) for 4 h. After the removal of the medium, RT-7 cells were stimulated with *P. gingivalis* LPS (1 *μ*g/mL) for 24 h. The IL-8 level in culture supernatants was determined by enzyme-linked immunosorbent assay and expressed as the mean ± standard deviations of three independent experiments. ^∗^*P* < 0.05 and ^∗∗^*P* < 0.01 compared to that in the control (no pretreatment with the *J. effusus* water extract or EGCG).

Subsequently, we examined whether pretreatment with the *J. effusus* L. water extract or EGCG inhibited IL-8 production in RT-7 cells stimulated with *P. gingivalis* LPS. 4 h pretreatment with the *J. effusus* L. water extract more effectively reduced IL-8 production in *P. gingivalis* LPS-stimulated RT-7 cells than in cells pretreated with EGCG (Figure 2).

### 3.3. Effects of *J. effusus* L. Water Extract on Chemokine Production in *P. gingivalis* LPS-Stimulated Oral Keratinocytes

**Figure 3:**
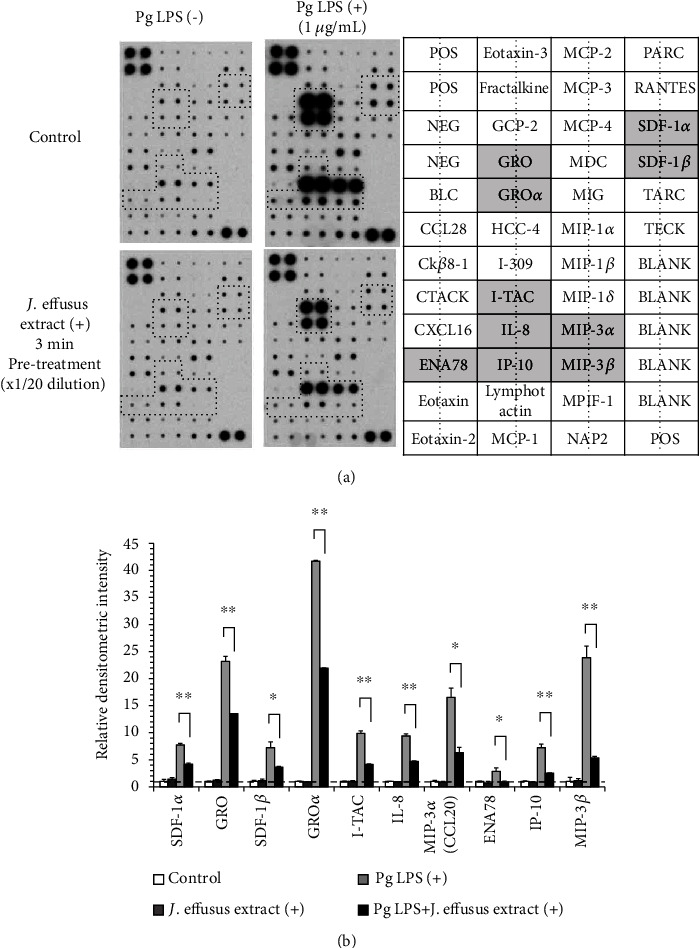
Effects of the *Juncus effusus* water extract on chemokine production in *Porphyromonas gingivalis* lipopolysaccharide- (LPS-) stimulated oral keratinocytes. RT-7 cells were pretreated with the *J. effusus* water extract (20-fold dilution) for 3 min. After the removal of the medium, RT-7 cells were cultured with *P. gingivalis* LPS (1 *μ*g/mL) for 24 h. The detection of chemokines in cell culture supernatants was performed using the Human Chemokine Antibody Array 1. (a) The results shown are representative images of two independent experiments with similar results. (b) A densitometric analysis of various chemokine productions. Bars indicate the relative densitometric intensities after the values were normalized with both positive and negative controls and background. In particular, positive controls were used to normalize the values from different membranes being compared. Values represent the means ± standard deviations of two independent experiments. ^∗^*P* < 0.05 and ^∗∗^*P* < 0.01 show significant differences between the indicated groups.

Considering actual clinical applications, it is impossible to hold the *J. effusus* L. water extract in the mouth for long periods of time. Next, we examined the changes in the levels of multiple chemokine products in RT-7 cells treated with *P. gingivalis* LPS for 24 h after 3 min pretreatment with *J. effusus* L. water extract using a chemokine antibody array. The levels of various chemokine products (stromal cell-derived factor- (SDF-) 1*α*, SDF-1*β*, growth-regulated oncogene (GRO), GRO*α*, interferon-inducible T-cell alpha chemoattractant (I-TAC), IL-8, macrophage inflammatory protein- (MIP-) 3*α*/CCL20, epithelial-derived neutrophil-activating peptide (ENA)78, interferon gamma-induced protein-10 (IP-10), MIP-3*β*) were increased more than twofold in RT-7 cells stimulated with *P. gingivalis* LPS for 24 h; however, the *J. effusus* L. water extract effectively inhibited all these chemokine products (Figure 3).

### 3.4. Effects of *J. effusus* L. Water Extract Pretreatment on IL-8 and CCL20 Production in TLR2 Ligand-Stimulated Oral Keratinocytes

**Figure 4:**
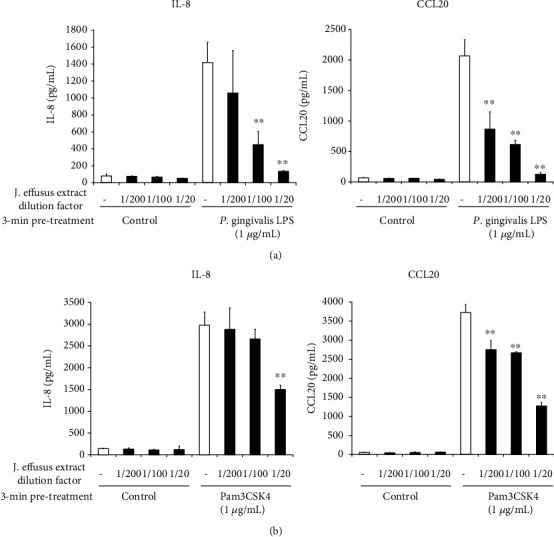
Effects of the *Juncus effusus* water extract pretreatment on interleukin-8 (IL-8) and C-C-chemokine ligand20 (CCL20) productions in Toll-like receptor2 ligand-stimulated oral keratinocytes. RT-7 cells were pretreated with the *J. effusus* water extract (20-, 100-, or 200-fold dilution) for 3 min. After the removal of the medium, RT-7 cells were stimulated with *Porphyromonas gingivalis* lipopolysaccharide or Pam3CSK4 (1 *μ*g/mL) for 24 h. IL-8 and CCL20 levels in culture supernatants were determined by enzyme-linked immunosorbent assay and expressed as the mean ± standard deviations of three independent experiments. ^∗∗^*P* < 0.01 compared to that in the control (no pretreatment with the *J. effusus* water extract).

Subsequently, we determined whether 3 min pretreatment with the *J. effusus* L. water extract can inhibit IL-8 and CCL20 production in RT-7 cells stimulated with the TLR2 ligand, including Pam3CSK4, a synthetic TLR2/TLR1 ligand. The 3 min pretreatment with the *J. effusus* L. water extract effectively reduced IL-8 and CCL20 production in *P. gingivalis* LPS- or Pam3CSK4-stimulated RT-7 cells in a concentration-dependent manner (Figure 4).

### 3.5. Inhibitory Effects of the *J. effusus* L. Water Extract on the Binding of TLR2/TLR1-Specific Ligand to Oral Keratinocytes

**Figure 5:**
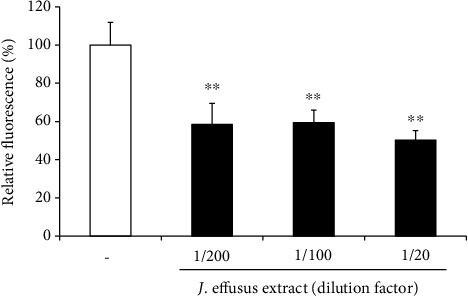
Inhibitory effects of the *Juncus effusus* water extract on the binding of Toll-like receptor (TLR)2/TLR1-specific ligand to oral keratinocytes. RT-7 monolayer was pretreated with 20-, 100-, or 200-fold diluted *J. effusus* water extract for 3 min. After the removal of the medium, RT-7 cells were treated with fluorescence-labeled Pam3CSK4 (1 *μ*g/mL) for 2 h at 37°C. After washing with phosphate-buffered saline for three times, the fluorescence level of Pam3CSK4 bound on RT-7 cells was measured with excitation at 485 nm and emission at 535 nm using a fluorescence microplate reader. Fluorescence levels shown on the *Y*-axis are relative to the binding efficiency of fluorescence-labeled Pam3CSK4 without *J. effusus* water extract pretreatment (control; 100%), and values are presented as the means ± standard deviations of five independent experiments. Asterisks indicate significant differences (^∗∗^*P* < 0.01) as compared with the control (no pretreatment with *J. effusus* water extract).

We investigated whether *J. effusus* L. water extract pretreatment could inhibit the binding of the TLR2/TLR1-specific ligand to RT-7 cells. 3 min pretreatment with the *J. effusus* L. water extract significantly inhibited the binding of fluorescence-labeled Pam3CSK4 to RT-7 cells. Moreover, 42.5% and 49.7% inhibitions of Pam3CSK4 binding were observed with 200- and 20-fold diluted *J. effusus* L. water extract pretreatments, respectively (Figure 5).

### 3.6. Effects of *J. effusus* L. Water Extract Pretreatment on IL-8 and CCL20 Production in TLR4 Ligand-Stimulated Oral Keratinocytes

**Figure 6:**
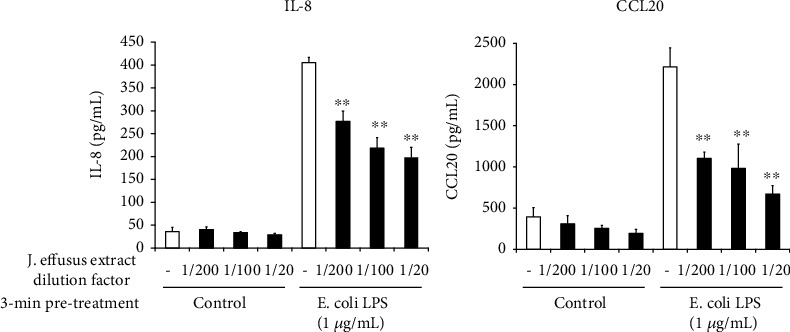
Effects of the *Juncus effusus* water extract pretreatment on interleukin-8 (IL-8) and C-C-chemokine ligand20 (CCL20) productions in Toll-like receptor4 ligand-stimulated oral keratinocytes. RT-7 cells were pretreated with the *J. effusus* water extract (20-, 100-, or 200-fold dilution) for 3 min. After the removal of the medium, RT-7 cells were stimulated with *Escherichia coli* lipopolysaccharide (1 *μ*g/mL) for 24 h. IL-8 and CCL20 levels in culture supernatants were determined by enzyme-linked immunosorbent assay and expressed as the mean ± standard deviations of three independent experiments. ^∗∗^*P* < 0.01 compared to the control (no pretreatment with the *J. effusus* water extract).

In addition to the TLR2 ligand, we also determined whether pretreatment with the *J. effusus* L. water extract can inhibit IL-8 and CCL20 production in RT-7 cells stimulated with the TLR4 ligand, *E*. *coli* LPS. 3 min pretreatment with the *J. effusus* L. water extract significantly reduced the production of IL-8 and CCL20 in *E*. *coli* LPS-stimulated RT-7 cells in a concentration-dependent manner (Figure 6).

### 3.7. Protective Effect of the *J. effusus* L. Water Extract Pretreatment against Chemical Irritation in Oral Keratinocytes

**Figure 7:**
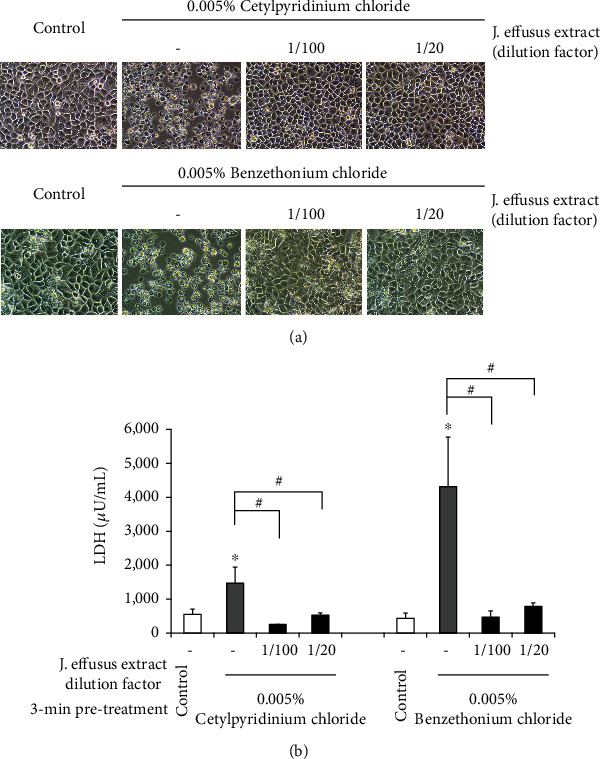
Protective effect of the *Juncus effusus* water extract pretreatment against chemical irritation to oral keratinocytes. RT-7 monolayers cultured in a 24-well plate were pretreated with a 100- or 20-fold diluted *J. effusus* water extract for 3 min at room temperature. After medium change, RT-7 monolayers were treated with 0.005% cetylpyridinium chloride (CPC) or 0.005% benzethonium chloride (BTC) for 24 h at 37°C. (a) Representative phase contrast micrographs of nonirritated control and CPC- or BTC-irritated RT-7 cells with or without *J. effusus* water extract pretreatment at ×20 magnification. The results are representative of five different experiments demonstrating similar results. (b) The levels of lactate dehydrogenase (LDH) in recovered cell culture supernatants were determined by an LDH cytotoxicity assay kit. Values represent the means ± standard deviations from 3 independent experiments. ^∗^*P* < 0.05 versus the nonirritated control. # (*P* < 0.05) shows significant differences between the indicated groups.

We determined whether pretreatment with the *J. effusus* L. water extract improved the shape of oral epithelial cells irritated by 0.005% CPC or 0.005% BTC (Figure 7(a)). Treatment with 0.005% CPC or 0.005% BTC caused RT-7 cells to deform and become round, resulting in the loss of cell adhesion and floating of RT-7 cells. In contrast, we observed that 3 min pretreatment with 100- or 20-fold diluted *J*. *effusus* L. water extract prior to CPC or BTC irritation protected cells from deforming and rounding.

We further confirmed this protective effect of *J. effusus* L. water extract pretreatment on CPC- or BTC-irritated RT-7 cells by measuring the released LDH level (Figure 7(b)). Irritation with 0.005% CPC or 0.005% BTC significantly increased the release of cellular LDH. In accordance with the cellular morphological observation, 3 min pretreatment with 100- or 20-fold diluted *J. effusus* L. water extract significantly decreased the released LDH level induced by CPC or BTC irritation to the control level.

### 3.8. The Inhibitory Effect of *J. effusus* L. Water Extract Pretreatment in the Presence of Antimicrobial Agents or Antifibrinolytics on IL-8 and CCL20 Production in TLR2 Ligand-Stimulated Oral Keratinocytes

**Figure 8:**
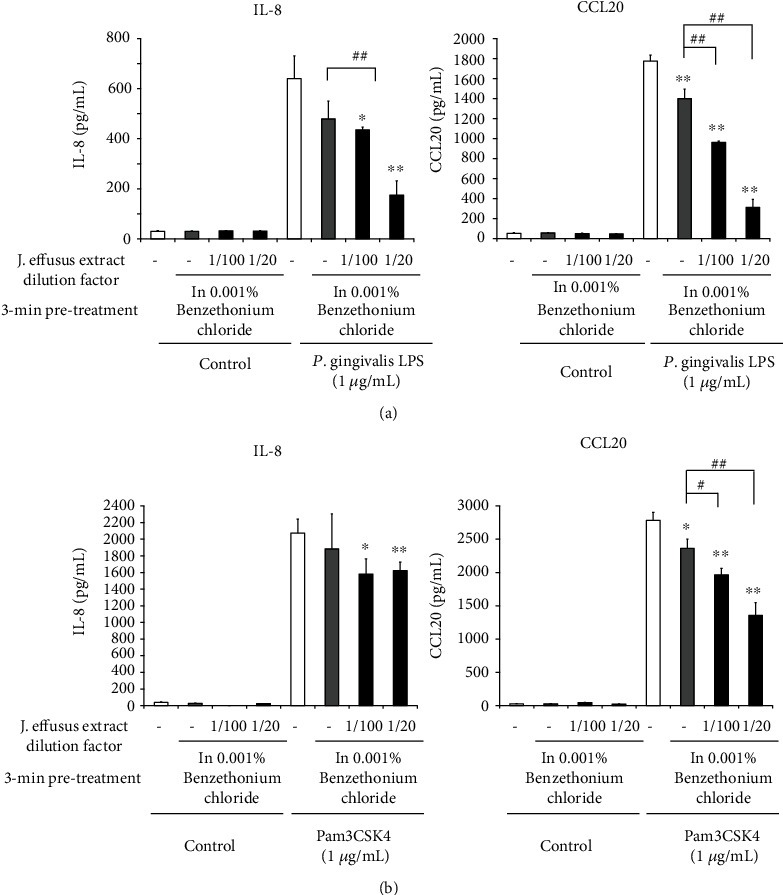
The inhibitory effect of the *Juncus effusus* water extract pretreatment on chemokine productions from Toll-like receptor2 ligand-stimulated oral keratinocytes in the presence of an antimicrobial agent. RT-7 cells were pretreated with the *J. effusus* water extract (20- or 100-fold dilution) and 0.001% benzethonium chloride for 3 min. After the removal of the medium, RT-7 cells were stimulated with (a) *Porphyromonas gingivalis* lipopolysaccharide or (b) Pam3CSK4 (1 *μ*g/mL) for 24 h. Interleukin-8 and C-C-chemokine ligand20 levels in culture supernatants were determined by enzyme-linked immunosorbent assay and expressed as the mean ± standard deviations of three independent experiments. ^∗^*P* < 0.05 and ^∗∗^*P* < 0.01 compared to that in the control (no pretreatment with the *J. effusus* water extract). # (*P* < 0.05) and ## (*P* < 0.01) show significant differences between the indicated groups.

**Figure 9:**
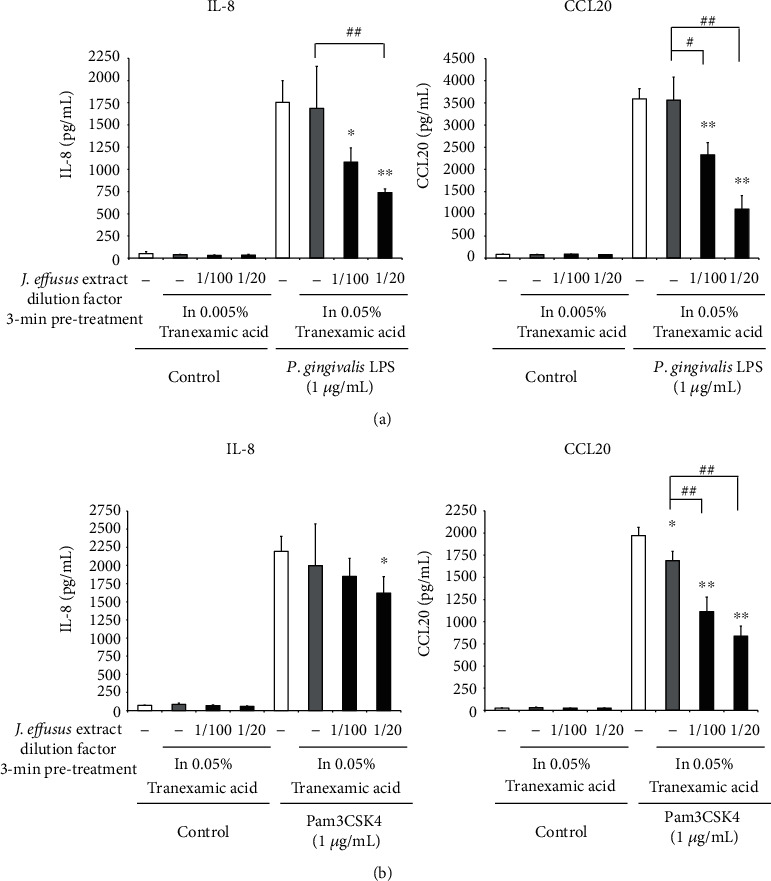
The inhibitory effect of the *Juncus effusus* water extract pretreatment on chemokine productions from Toll-like receptor2 ligand-stimulated oral keratinocytes in the presence of an antifibrinolytic. RT-7 cells were pretreated with the *J. effusus* water extract (20- or 100-fold dilution) and 0.05% TA for 3 min. After the removal of the medium, RT-7 cells were stimulated with (a) *Porphyromonas gingivalis* lipopolysaccharide or (b) Pam3CSK4 (1 *μ*g/mL) for 24 h. Interleukin-8 and C-C-chemokine ligand20 levels in culture supernatants were determined by enzyme-linked immunosorbent assay and expressed as the mean ± standard deviations of three independent experiments. ^∗^*P* < 0.05 and ^∗∗^*P* < 0.01 compared to the control (no pretreatment with the *J. effusus* water extract). # (*P* < 0.05) and ## (*P* < 0.01) show significant differences between the indicated groups.

TA, a synthetic analog of the amino acid lysine, impedes the proteolytic degradation of fibrin by preventing the attachment of plasminogen and plasmin, and this antifibrinolytic agent can be used as a mouthwash and toothpaste to prevent gingival bleeding, as well as postsurgical bleeding in patients on anticoagulants after oral surgery [34]. We further examined whether pretreatment with the *J. effusus* L. water extract in the presence of BTC as an antimicrobial agent or TA as an antifibrinolytic can inhibit IL-8 and CCL20 production in TLR2 ligand-stimulated oral keratinocytes. In the presence of BTC or TA, 3 min pretreatment with the *J. effusus* L. water extract significantly reduced the production of IL-8 and CCL20 from Pam3CSK4- or *P. gingivalis* LPS-stimulated RT-7 cells in a concentration-dependent manner (Figures 8 and 9). These results suggest that the *J. effusus* L. water extract has anti-inflammatory effects even when mixed with mouthwash containing pharmacological agents, such as antimicrobial agents and antifibrinolytics.

## 4. Discussion

Periodontitis is a chronic infectious inflammatory disease that occurs in response to the development of dental plaque biofilm, which leads to periodontal tissue destruction and finally tooth loss. Gingival epithelial cells, as the first physical barrier, are in the forefront in fighting the bacterial attack and regulate the local innate immune reaction against biofilm-formed pathogens, such as *P. gingivalis* [35]. Chemokines produced from the gingival epithelium trigger and enhance acute periodontal inflammation and recruit and activate various important effector cells during periodontal disease. In particular, IL-8 attracts neutrophils, a major contributor to tissue damage during inflammatory diseases, and enhances neutrophil extracellular trap formation, and CCL20 also links innate and adaptive immune responses by chemoattracting and activating immature dendritic cells. Both chemokines are extremely positive in inflamed gingival epithelial cells [13, 36]. Many previous studies have focused on an effective chemotherapeutic strategy in killing the causative bacteria to prevent periodontal infection; however, the overuse of antibiotics as chemicals causes the emergence of highly resistant and virulent strains of microorganisms, which is becoming a serious social problem in the medical field [37, 38]. Therefore, the development of a therapy for the suppression of overproduction of proinflammatory mediators, such as IL-8 and CCL20, is also necessary.

*J. effusus* L. is an herbaceous perennial belonging to the Juncaceae family and is distributed in marshlands, such as rice paddies and coastal marshes in Japan and China. The dried medullae of *J. effuses* L. that have antipyretic, antiphlogistic, and sedative effects are used as crude and traditional medicines in both countries [39, 40].

In this study, we used the *J. effusus* L. water extract as a natural product, demonstrating that the *J. effusus* L. water extract can inhibit IL-8 and CCL20 production in oral keratinocytes, RT-7 cells, stimulated with *P. gingivalis* LPS, and the efficacy of the 20-fold diluted extract was comparable to that of EGCG at 50 *μ*g/mL (Figure 1). It is interesting to note that 4 h pretreatment with *J. effusus* L. water extract reduced IL-8 production more effectively in *P. gingivalis* LPS-stimulated RT-7 cells than in cells pretreated with EGCG (Figure 2). Regarding the signaling activated via cell surface receptor of EGCG, previous studies reported that biological activities of EGCG are mediated through binding to the cell surface 67 kDa laminin receptor [41, 42] that is highly associated with lipid rafts. Lipid rafts are well-known platforms for immunomodulative signal transduction and membrane traffic pathways and therefore can regulate membrane function to mediate various cellular functions of EGCG in eukaryotic cells [43]. Moreover, 3 min pretreatment with the *J. effusus* L. water extract effectively inhibited the production of various chemokines (SDF-1*α*, SDF-1*β*, GRO, GRO*α*, I-TAC, IL-8, MIP-3*α*/CCL20, ENA78, IP-10, and MIP-3*β*) in RT-7 cells stimulated with *P. gingivalis* LPS for 24 h (Figure 3). These findings suggest that the *J. effusus* L. water extract has an anti-inflammatory effect on oral keratinocytes stimulated with endotoxin from periodontal pathogens and may have therapeutic potential for the treatment of periodontal inflammation. In addition, 3 min pretreatment with the *J. effusus* L. water extract effectively reduced IL-8 production in *P. gingivalis* LPS-stimulated RT-7 cells in a concentration-dependent manner (Figure 4). These results also suggest that the inhibition mechanism of the *J. effusus* L. water extract is likely different from that of EGCG. We observed that 3 min pretreatment with the *J. effusus* L. water extract inhibited the binding of fluorescence-labeled Pam3CSK4, a TLR2/TLR1-specific ligand, to RT-7 cells (Figure 5). Moreover, this 3 min pretreatment could reduce the inflammatory reaction of other bacterial PAMPs and *E*. *coli* LPS, as a TLR4 ligand (Figure 6). These findings imply that pretreatment with the *J. effusus* L. water extract reduces the innate immune response by blocking the binding sites on oral epithelial cells for TLR2 and TLR4 ligands, suggesting that the *J. effusus* L. water extract may be beneficial in the prevention of innate immune responses that leads to periodontal tissue inflammation. It has been reported that EGCG inhibits intracellular signal transduction pathways and ultimately suppresses the transcription of various inflammation-related mediators [44–46]. This study indicated that pretreatment with *J. effusus* L. water crude extract suppressed the expression and production of chemokine genes by inhibiting the binding of TLR ligands. A detailed analysis of this suppression mechanism is currently under investigation.

With the development of new oral care formulations for the prevention of oral infectious diseases, such as periodontitis, the products include active and excipient ingredients and may increase the risk of causing oral irritation in some populations, such as the elderly [47]. Quaternary ammonium compounds, CPC and BTC, are commonly used in antiseptic mouthwashes or rinses for oral health care to prevent oral infectious diseases. Although these compounds seem to be safer than chemically active disinfectants, such as glutaraldehyde, there is a concern that these compounds can cause mucosal irritation and have cytotoxic effects on human cells, such as keratinocytes [48]. We observed that 3 min pretreatment with a 100-fold diluted *J*. *effusus* L. water extract can protect oral epithelial cells by CPC or BTC irritation using both microscopic observation and the cytotoxicity assay by measuring the released LDH level (Figure 7), indicating that the *J*. *effusus* L. water extract has beneficial effects on maintaining the barrier function of oral epithelial cells and may be a promising active ingredient in a mouthwash. We also clarified that treatment with *J*. *effusus* L. water extract makes the surface of the glass plate and saliva-treated hydroxyapatite plate superhydrophilic (unpublished data). Therefore, it is speculated that the attachment of proteins and the stimulation of chemical components can be suppressed by changing the surface property of epithelial cells to superhydrophilic by pretreatment with *J*. *effusus* L. water extract.

In addition to disinfectants, mouthwashes and toothpastes contain antifibrinolytic agents, such as TA, to prevent prolonged bleeding of the gingiva [34]. Even in the presence of BTC or TA, 3 min pretreatment with the *J*. *effusus* L. water extract significantly reduced chemokine secretion from Pam3CSK4- or *P*. *gingivalis* LPS-stimulated RT-7 cells in a concentration-dependent manner (Figures 8 and 9), suggesting that the *J. effusus* L. water extract can exert anti-inflammatory effects even when mixed with a mouthwash containing pharmacological agents, such as antimicrobial agents and antifibrinolytics.

Previous phytochemical studies have shown that the traditional Chinese medicine of *J*. *effuses* contains considerable amounts of bioactive constituents, such as phenanthrenes, coumaric acid, flavones, cycloartanes, coumaroyl glycerides, and dihydrodibenzoxepins [40, 49]. The plant materials (stems, leaves, medullae, tubers, roots, rhizomes, or whole plants) have been extracted usually with ethyl acetate, acetone, methanol, ethanol, and aqueous ethanol at room temperature [50]. The fractions from ethanol extracts of medullae and root bark of *J*. *effuses*, such as dehydroeffusol, have anticancer, anti-inflammatory, and apoptosis-protective activities [15, 49, 51–54]. Previous studies reported that phenanthrenes isolated from methanol extract of *J. inflexus* roots are the main bioacative components exhibiting antimicrobial activity against several pathogens, such as methicillin-resistant *Staphylococcus aureus*, and anti-inflammatory and antioxidant effects [55, 56]. A recent study has reported that phenanthrenes, compounds isolated from *J*. *effuses*, can significantly inhibit superoxide anion generation and elastase release in a fMLP/CB-induced human neutrophilic inflammation model [57]. Despite the concern that ethanol can potentially increase the risk of oral cancer, many mouthwashes contain more than 80% ethanol, which is not crucial for preventing and reducing gingivitis caused by dental plaque [58]. Therefore, the development of ethanol-free mouthwashes with biological activities, such as anti-inflammatory and antimicrobial effects, is needed. In the present study, we investigated the anti-inflammatory and protective effects of extracts with water, not organic solvents, from *Juncus effuses* on oral keratinocytes. Regarding the prevention of oral inflammation elicited by bacterial infection, this study demonstrated that the anti-inflammatory activity of the *J*. *effusus* L. water extract was the same or higher than that of EGCG. Considering that oral care contributes to systemic health, we infer that clinical applications of the *J*. *effusus* L. water extract with protective effects in the oral mucosa, as well as an anti-inflammatory activity, are also useful in immunocompromised hosts and elderly people. However, the question remains as to which components in *Juncus effusus* L. water extract are important in these effects on oral keratinocytes. We speculate that active components in *Juncus effusus* L. water extract other than phenanthrenes isolated with methanol from the *Juncus* family may be included. The isolation, identification, and characterization of the active components from *Juncus effusus* L. water extract showing an anti-inflammatory activity and clarification of their detailed action mechanisms are now under investigation, and these may enable to prevent oral infections such as periodontal infections and to maintain oral epithelial function. In addition, considering a case of the use of *Juncus effusus* L. water extract as general mouthwash, we need to determine the effects of this extract in a shorter time pretreatment period like few seconds, and they are now under investigation. Furthermore, based on these additional experimental results, a clinical study using this extract as mouthwash is expected.

## 5. Conclusion

Our study showed the effects of the water extract from *J. effusus* L. on the inhibition of the inflammatory reaction elicited by bacterial infection and protection of the oral epithelium by chemical irritation. Pretreatment of oral epithelial cells with the water extract from *J. effusus* L. significantly reduced IL-8 and CCL20 production elicited by stimulation with *P. gingivalis* LPS in a concentration-dependent manner with effects comparable to or more than those of EGCG and protected oral epithelial cells from injury by chemical irritation with CPC and BTC. Furthermore, the water extract from *J*. *effusus* L. in the presence of an antimicrobial agent, BTC, or antifibrinolytic, TA, used as ingredients in mouthwashes, could significantly reduce IL-8 and CCL20 production in *P. gingivalis* LPS- or Pam3CSK4-stimulated oral epithelial cells in a concentration-dependent manner. These findings may also lead to the clinical application of the *J*. *effusus* L. water extract as an oral care product for the prevention of oral infections, such as periodontitis, and for maintenance of oral epithelial function.

## Data Availability

The data sets are available on request.
